# Synergistic MDM2-STAT3 Inhibition Demonstrates Strong Anti-Leukemic Efficacy in Acute Lymphoblastic Leukemia

**DOI:** 10.3390/ijms26178648

**Published:** 2025-09-05

**Authors:** Erhan Aptullahoglu, Emrah Kaygusuz

**Affiliations:** 1 Department of Molecular Biology and Genetics, Faculty of Science, Bilecik Şeyh Edebali University, 11100 Bilecik, Türkiye; emrah.kaygusuz@bilecik.edu.tr; 2 Biotechnology Application and Research Centre, Bilecik Şeyh Edebali University, 11100 Bilecik, Türkiye

**Keywords:** acute lymphoblastic leukemia (ALL), MDM2, STAT3, RG7388 (idasanutlin), BBI608 (napabucasin), targeted cancer therapies

## Abstract

Acute lymphoblastic leukemia (ALL) remains a formidable therapeutic challenge, particularly within high-risk cohorts. Advances in next-generation sequencing have elucidated critical mutations that significantly influence prognosis and therapeutic decision-making. Tyrosine kinase inhibitors (TKIs) have significantly improved treatment outcomes in Philadelphia chromosome-positive (Ph+) ALL. Meanwhile, emerging therapies such as monoclonal antibodies and chimeric antigen receptor (CAR) T-cell therapies show promise for B-cell ALL, although they are associated with considerable toxicities. These developments underscore the persistent need for alternative therapeutic strategies that can benefit a wider range of patients. In this study, human ALL cell lines—characterized by either wild-type or mutant *tumor protein p53 (TP53)* status—were treated with RG7388 (an MDM2 (mouse double minute 2 homolog) inhibitor) and BBI608 (a STAT3 (signal transducer and activator of transcription 3) inhibitor), both as single agents and in combination. Cell viability was quantified using XTT assays, while apoptosis was assessed via flow cytometry. Additionally, immunoblotting and qRT-PCR were employed to evaluate changes in protein and gene expression, respectively. RG7388 demonstrated potent growth inhibition in the majority of ALL cell lines, with p53-mutant cell lines exhibiting resistance. BBI608 reduced cell viability across all tested cell lines, though with variable sensitivity. Notably, the combination of RG7388 and BBI608 elicited synergistic anti-proliferative effects in p53 wild-type and partially functional p53-mutant cells, enhancing apoptosis and stabilizing p53 protein levels. In contrast, MOLT-4 cells, which harbor concurrent *TP53* and *STAT3* mutations, did not benefit from the combination treatment, indicating an inherent resistance phenotype within this subset. Collectively, these findings highlight the therapeutic potential of combined MDM2 and STAT3 inhibition in ALL, particularly in p53 wild-type and partially functional p53-mutant contexts. This combinatorial approach augments apoptosis and tumor growth suppression, offering a promising avenue for expanding treatment options for a broader patient population. Further investigation is warranted to validate these preclinical findings and to explore translational implications in genetically diverse ALL subsets.

## 1. Introduction

Acute lymphoblastic leukemia (ALL) is a complex hematological malignancy characterized by a wide range of clinical features and variable treatment responses [1]. Despite significant advancements in chemotherapy and targeted therapies, high-risk groups such as those with myeloid mutations or Philadelphia chromosome-positive (Ph+) ALL often experience poor prognosis and treatment resistance [2,3], underscoring the need for more effective therapeutic strategies. Current treatments include chemotherapy [4,5], radiotherapy [6], immunotherapy [7,8], targeted therapies [9], and stem cell transplantation [10,11], with remission as the primary goal of initial therapy.

While these treatment modalities have significantly advanced the management of ALL, each carries important limitations that restrict their long-term efficacy [12,13]. Tyrosine kinase inhibitors (TKIs), such as imatinib and dasatinib, have markedly improved outcomes in Ph+ ALL and are now a standard of care in this subset [9]. Nevertheless, their clinical benefit is frequently undermined by the emergence of resistance mutations, particularly the ABL1 T315I gatekeeper mutation, and by relapse despite initial responses [14]. Monoclonal antibodies, including blinatumomab (a bispecific T-cell engager targeting CD19 and CD3) [15,16] and inotuzumab ozogamicin [17,18] (an anti-CD22 antibody-drug conjugate), have broadened therapeutic options in relapsed or refractory B-ALL. However, their use is constrained by severe treatment-related toxicities—cytokine release syndrome for blinatumomab [19], hepatotoxicity and veno-occlusive disease for inotuzumab [20,21]—as well as by the emergence of antigen-negative escape variants that limit durability of response [22,23]. Chimeric antigen receptor (CAR) T-cell therapies, exemplified by tisagenlecleucel, have produced unprecedented remission rates of up to 80% in pediatric and young adult B-ALL, representing a paradigm shift in immunotherapy [8]. Yet, these benefits are tempered by life-threatening toxicities [24], high cost, logistical complexity, and relapse driven by antigen loss or poor CAR T-cell persistence [25]. Collectively, these limitations highlight that although innovative therapies have improved short-term outcomes, their curative potential remains limited by resistance, toxicity, and disease recurrence. Thus, there is an urgent need for alternative therapeutic approaches that act through distinct molecular mechanisms and can be applied across genetically heterogeneous ALL populations. In this context, enzyme therapies such as L-asparaginase, which have long been integral to ALL treatment, merit mention. L-asparaginase induces leukemic cell apoptosis by depleting extracellular asparagine, which is a critical nutrient for leukemic blasts [26]. However, its clinical utility is frequently limited by hypersensitivity reactions, hepatotoxicity, pancreatitis, and the development of resistance via upregulation of asparagine synthetase [27]. Moreover, its low tumor selectivity may affect normal tissues, contributing to systemic toxicity [27]. In contrast, the proposed strategy of combined MDM2 and STAT3 inhibition offers a more targeted, non-enzymatic approach that seeks to restore p53 function and inhibit STAT3-driven oncogenic signaling. This dual targeting may provide enhanced selectivity, reduced toxicity, and greater efficacy, particularly in cases of relapsed or treatment-resistant ALL.

The *TP53* gene, which encodes the tumor suppressor protein p53, plays a critical role in maintaining cellular homeostasis by regulating processes such as DNA repair, apoptosis, and cell cycle arrest [28]. Mutations in *TP53* are common in various cancers [29], particularly more frequently observed in solid tumors, where they are detected in approximately 50% of cases across all cancer types [30]. In contrast, *TP53* mutations are less frequent in hematological cancers like ALL, occurring in about 15% of cases at diagnosis [31,32] and up to 45% at relapse [33,34]. *TP53* mutations in ALL are generally associated with poor prognosis and reduced treatment efficacy [35]. In some cases, even when *TP53* is wild-type, which is typically associated with functional p53, its activity can be suppressed by various factors, including the overexpression of MDM2, a negative regulator of p53 [36]. This opens the possibility of targeting MDM2 to reactivate p53 in cancers with wild-type *TP53* [37,38].

MDM2 is an E3 ubiquitin ligase that binds to p53, inhibiting its activity and promoting its degradation [36]. Cellular stress triggers post-translational modifications of p53, leading to the disruption of the p53-MDM2 interaction. This results in the release and accumulation of p53, which then activates its target genes involved in cell cycle arrest, apoptosis, or senescence [39]. Overexpression of MDM2 contributes to reduced p53 activity in various cancers [40], including ALL [41]. Nutlin-3 was the first selective MDM2-p53 binding antagonist demonstrated to activate p53 and its downstream signaling in preclinical models [42]. Subsequently, several next-generation MDM2 inhibitors, including RG7388 [43], HDM201 [44], AMG232 [45], and others [46,47,48], have been developed. These compounds have shown efficacy in reducing cancer cell viability and proliferation in preclinical models, including those of leukemia and lymphoma [49,50,51,52,53,54,55,56,57]. Currently, many of these inhibitors are being evaluated in clinical trials, both as monotherapies [58,59] and in combination treatments [60,61,62]. In certain cases, such as patients exhibiting multiple mutations or experiencing relapses, resistance may arise when monotherapy is employed [63,64]. Various efforts have been made to understand the mechanisms of resistance [65,66]; however, from a broader perspective, the search for combination therapy strategies remains a key focus in targeted treatments.

STAT3, a member of the STAT protein family, is a key regulator of cell proliferation, differentiation, and apoptosis [67]. Under normal conditions, the cellular activation of STAT3 is rapid and transient [68]. STAT3 is frequently aberrantly hyperactivated in both cancerous and non-cancerous cells within the tumor microenvironment, driving the production of immunosuppressive factors, promoting uncontrolled cancer cell proliferation, and inhibiting apoptosis [69,70]. This persistent activation of STAT3 is a hallmark of leukemia and contributes to tumor progression [71]. For these reasons, targeting STAT3 with small molecule inhibitors has become an attractive therapeutic strategy for leukemia treatment, showing promising results in preclinical studies [72].

The objective of this study is to investigate a novel therapeutic strategy for ALL by combining an MDM2 inhibitor with a STAT3 inhibitor. While this combinatorial approach has been explored in chronic lymphocytic leukemia [73], it has not yet been studied in ALL. The rationale is based on the premise that STAT3 activation plays a key role in suppressing the function of p53, a critical tumor suppressor protein [74]. By targeting both MDM2 and STAT3, we hypothesize that p53 can be reactivated while simultaneously inhibiting STAT3-driven tumor growth, thereby promoting enhanced apoptosis in ALL cells. Additionally, STAT3 is frequently activated in leukemia, often independent of p53 status, making it an attractive therapeutic target. The rationale for this strategy is based on the idea that reversing the suppression of p53 by STAT3, as well as inhibiting STAT3 activation itself, could lead to significant tumor growth inhibition and increased cell death. Moreover, non-genotoxic MDM2 inhibitors, which activate p53 without damaging healthy cells [49], offer a distinct advantage that we aim to capitalize on in combination with STAT3 inhibition. Increased STAT3 activation is observed not only in wild-type p53 cells but also in cancer cells harboring p53 mutations [75,76]. Targeting STAT3 has been shown to be an effective strategy for inhibiting the growth of aggressive cancer cells exhibiting increased STAT3 Tyr705 phosphorylation and p53 mutations [77]. Furthermore, the potential regrowth of *TP53* mutant subclones, which contribute to variable responses to MDM2 inhibitors and disease relapse [78,79], could be effectively suppressed with this combined therapeutic strategy.

Given that both MDM2 and STAT3 inhibitors have demonstrated promise in preclinical cancer studies, including those involving other hematologic malignancies, combined targeting of these two pathways may offer a more effective and safer treatment approach for ALL. Through this research, we aim to explore the therapeutic potential of this strategy, providing a novel direction for improving treatment outcomes in ALL patients, particularly those with limited therapeutic options.

## 2. Results

### 2.1. The Inhibition of Acute Lymphoblastic Leukemia Cell Proliferation by Napabucasin and Idasanutlin

**Figure 1:**
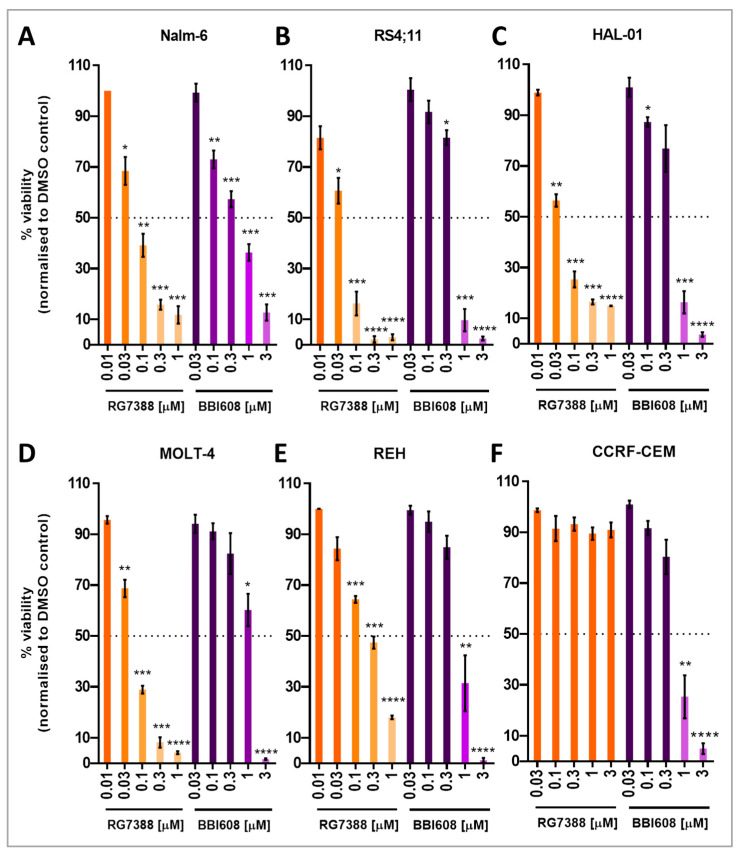
Dose-dependent inhibition of cell viability by RG7388 and BBI608 across six different ALL cell lines. The cell lines Nalm-6 (**A**), RS4;11 (**B**), HAL-01 (**C**), MOLT-4 (**D**), REH (**E**), and CCRF-CEM (**F**) were treated with increasing doses of RG7388 (0.01 to 1 µM, up to 3 µM for CCRF-CEM) and BBI608 (0.03 to 3 µM). After 72 h of drug treatment, metabolic activity was assessed using the XTT assay. Cell viability data were normalized to the DMSO control (without drug) and are presented as percentages. Data represent the mean ± standard error of the mean (SEM) from at least three independent experiments. For each cell line, each dose was statistically compared to its corresponding control using a paired *t*-test, with significant differences indicated on the graph. * *p* < 0.05; ** *p* < 0.01; *** *p* < 0.001; **** *p* < 0.0001.

Before investigating the combined effects, six different ALL cell lines were individually exposed to escalating concentrations of the MDM2 inhibitor RG7388 and the STAT3 inhibitor BBI608. Cell viability was assessed using the XTT assay 72 h post-treatment, with results compared to the DMSO solvent control (0 nM). RG7388 induced growth inhibition in five out of the six ALL cell lines, all of which exhibited IC50 values within the submicromolar range (47–289 nM; Figure 1 and Table 1). In contrast, the CCRF-CEM cell line, which harbors two missense mutations in the DNA-binding domain of the *TP53* gene, displayed resistance to RG7388 (Figure 1 and Table 1). No significant effects were observed in CCRF-CEM cells with the *TP53* double mutation, even at concentrations up to 3 µM (Figure 1 and Table 1). The REH cell line, carrying the heterozygous c.541C>T mutation in the *TP53* gene (Table 1), demonstrated a slightly elevated IC50 compared to the other sensitive cell lines. However, despite the increased IC50, the mutant p53 REH cells remained responsive to treatment within the drug-sensitive range.

BBI608 was also tested independently and induced growth inhibition in all cell lines, with IC50 values ranging from 562 to 1114 nM (Table 1). Cells generally tolerated doses below 0.3 µM with high viability, whereas suppression of STAT3 activity at higher concentrations led to a significant decrease in viability. Although a slightly increased IC50 was observed in MOLT-4 cells, which harbor heterozygous missense mutations in the *STAT3* gene and are the only *STAT3* mutant cells in the panel, no statistically significant increase in IC50 was detected in these cells compared to the other cell lines (Table 1).

### 2.2. MDM2 Inhibition Exhibits Synergistic Antiproliferative Effects with STAT3 Inhibitor In Vitro

**Figure 2:**
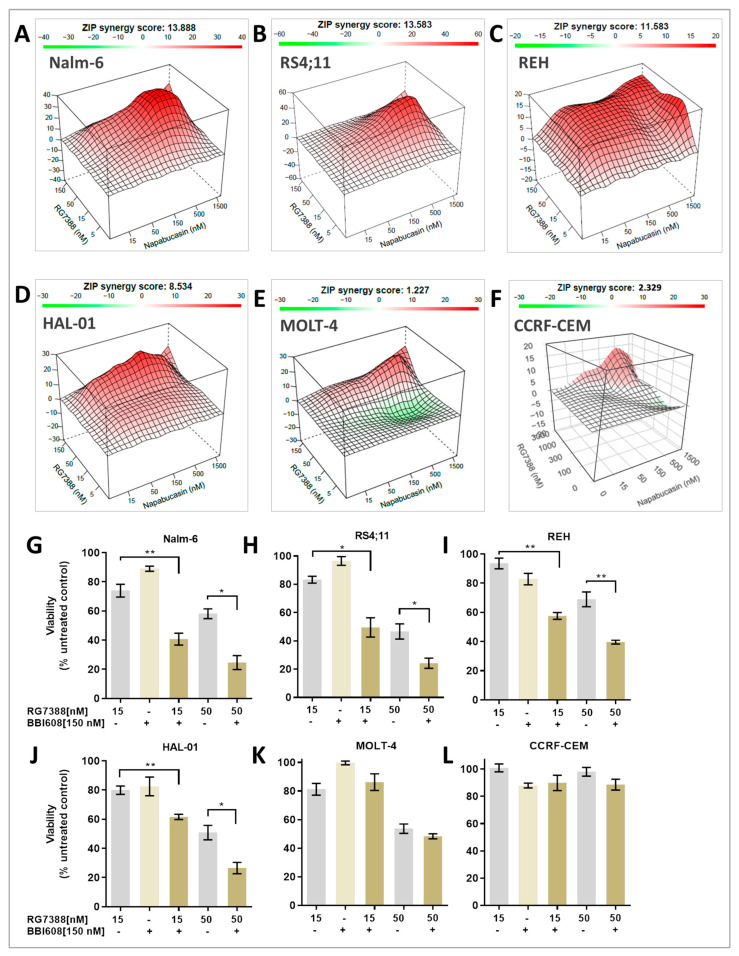
Synergistic effect of RG7388 and BBI608 on ALL cell lines. Synergy heat maps for drug combinations were generated using the zero interaction potency (ZIP) model for the cell lines Nalm-6 (**A**), RS4;11 (**B**), REH (**C**), HAL-01 (**D**), MOLT-4 (**E**), and CCRF-CEM (**F**). Panels (**G**–**L**) show cell viability as a percentage of the untreated control for each cell line after 72 h of treatment with 150 nM BBI608, with or without the indicated concentrations of RG7388 (15 or 50 nM). Significant differences between treatments were assessed using a paired *t*-test (* *p* < 0.05; ** *p* < 0.01). Data are presented as the mean ± standard error of the mean (SEM) from at least three independent experiments.

The effects of the combination treatment were investigated using a concentration-response matrix assay to assess the synergistic interactions across various ALL cell lines. Growth inhibition following treatment with the MDM2 inhibitor RG7388, either alone or in combination with BBI608, was evaluated in both p53 wild-type (p53^WT^) and p53 mutant (p53^MUT^) ALL cell lines, including Nalm-6, RS4;11, and HAL-01 for p53^WT^, and MOLT-4, REH, and CCRF-CEM for p53^MUT^. The combination of BBI608 and RG7388 significantly enhanced growth inhibition in all p53^WT^ cell lines, as well as in the p53^MUT^ REH cells (Figure 2A–D,G–J), which harbor a single mutation in the DNA-binding domain of *TP53* and exhibit sensitivity to MDM2 inhibition (Table 1).

The combination drug experiments yielded striking results, revealing synergy in four p53^WT^ cell lines (REH, RS4;11, Nalm-6, and HAL-01). The delta synergy scores for these cell lines ranged from 8.5 to 13.9, while the highest synergy scores observed in the combination treatment heat map ranged from 13 to 25.7 (Table 2). In contrast, the MOLT-4 cells, which harbor two distinct mutations in the *STAT3* gene and likely possess disrupted STAT3 activity, showed a synergy score of 1.2, indicating an additive interaction rather than synergy (Figure 2E,K; Table 3). The CCRF-CEM cell line, which carries two missense mutations in the DNA-binding domain of the *TP53* gene, exhibited resistance to RG7388 treatment (Table 1). When combined with the STAT3 inhibitor, there was no significant reduction in cell viability, and no notable benefit was observed from the combination therapy in this cell line (Figure 2F,L; Table 2).

### 2.3. Suppression of STAT3 Activity Amplifies P53 Downstream Activation Induced by MDM2 Inhibition

**Figure 3:**
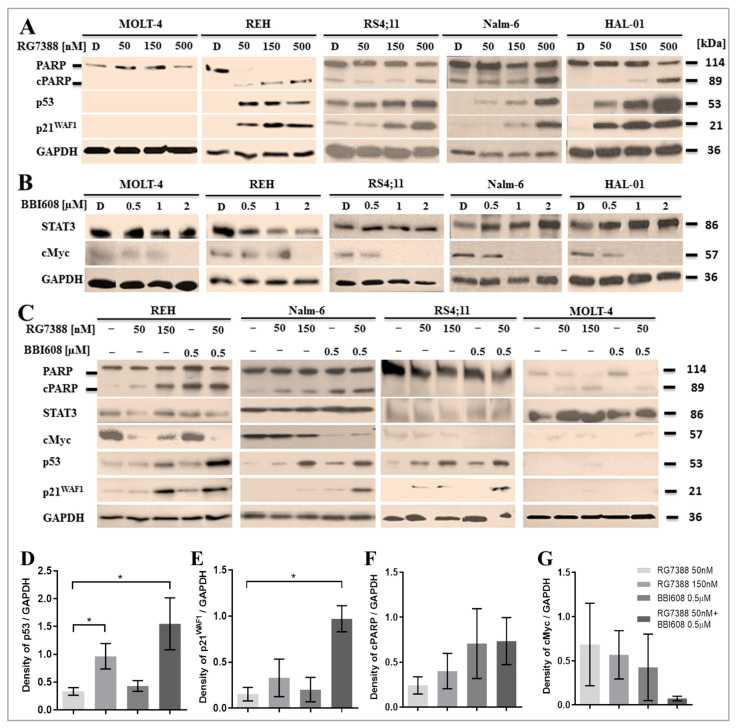
(**A**) Immunoblotting images following RG7388 treatment in ALL cell lines. MOLT-4, REH, RS4;11, Nalm-6, and HAL-01 cell lines were treated with RG7388 at concentrations of 0, 50, 150, and 500 nM for 24 h. p53 stabilization and expression of p21^WAF1^, a key marker of p53 functionality, were assessed. A decrease in full-length PARP and an increase in cleaved PARP (cPARP) were observed, which are well-established markers of apoptosis. (**B**) Effect of BBI608 on STAT3 downstream signaling in ALL cell lines. After 48 h of treatment with different concentrations of BBI608 (0, 0.5, 1, and 2 µM), the inhibition of cMyc, a downstream target of the STAT3 pathway, was observed in ALL cells. (**C**) Comparative analysis of protein levels following single and combined treatments. Protein levels of full-length/cleaved PARP, STAT3, cMyc, p53, and p21^WAF1^ were assessed 24 h after treatment with RG7388 (50 and 150 nM), 0.5 µM BBI608, or both drugs in combination for REH, Nalm-6, RS4;11, and MOLT-4 cell lines. Induction of p21^WAF1^ supports functional p53 activity. No p53 stabilization or p21^WAF1^ induction was detected in MOLT-4 cells, which harbor mutations in *TP53* and *STAT3* genes. GAPDH was used as a loading control. The red dashed lines indicate the cropped blots run on the same gel. Densitometric analysis of p53 (**D**), p21^WAF1^ (**E**), cleaved PARP (**F**), and cMyc (**G**) protein levels in REH, Nalm-6, and RS4;11 cell lines following treatment with RG7388, BBI608, or their combination. Protein expression levels were normalized to GAPDH as a loading control. Data are presented as mean ± SD, with error bars representing the variation among the three cell lines. Statistical significance was determined using a paired *t*-test (* *p* < 0.05). nM: nanomolar; µM: micromolar.

Following treatment with RG7388, p53 protein levels and the expression of its transcriptional target, p21^WAF1^, were assessed by immunoblotting in ALL cell lines. In p53^WT^ cell lines exposed to increasing concentrations of RG7388 (0, 50, 150, and 500 nM), dose-dependent stabilization of p53 was clearly observed in all p53^WT^ cells, including RS4;11, Nalm-6, and HAL-01 (Figure 3A). This stabilization of p53 was concomitant with a corresponding increase in the downstream transcriptional target p21^WAF1^, reflecting functional p53 stabilization. These results confirm the target-specific effects of RG7388.

Additionally, RG7388 treatment for 24 h resulted in a dose-dependent increase in the concentration of the 89 kDa cleaved PARP (cPARP), the cleaved product of full-length PARP. The elevation in cPARP levels is indicative of apoptosis, as PARP-1 is a well-known substrate of caspases, and its cleavage is an established marker of apoptosis [80].

Regarding the *TP53* gene status of the MOLT-4 cell line, various *TP53* mutations, including an undefined splicing mutation, have been reported. Mutations such as p.R248Q, p.L111V, p.R306*, or the absence of mutations have been described in the literature. According to the COSMIC database, MOLT-4 carries the p.R306* mutation, while the ATCC cell bank reports the p.R248Q mutation. As shown in Figure 3A, no stabilization of p53 was observed in MOLT-4 cells following RG7388 treatment, which would normally induce p53 stabilization in functional cells due to MDM2 inhibition and the release of p53. Additionally, p21^WAF1^, a transcriptional target of p53, was undetectable in MOLT-4 cells, further supporting the presence of non-functional p53. In contrast, in the *TP53* mutant REH cell line, there was evident p53 stabilization, although a dose-dependent increase was not observed. Nevertheless, p21^WAF1^ was transcriptionally activated, indicating some level of functional p53 response.

The effects of BBI608, a selective STAT3 inhibitor that targets STAT3 phosphorylation and activation, were also evaluated in ALL cells. Immunoblotting revealed that BBI608 significantly reduced cMyc expression in ALL cells without significantly affecting total STAT3 protein levels (Figure 3B). At doses above the IC50 (≥1 µM), a marked decrease in cMyc was observed (Figure 3B). In MOLT-4 cells, which harbor heterozygous mutations in the *STAT3* gene, a stronger STAT3 signal and a lower cMyc signal were observed, suggesting partial loss of STAT3 phosphorylation (Figure 3B).

The effects of combination therapy on p53 stabilization and p21^WAF1^ upregulation were assessed in four different cell lines. In *STAT3* wild-type cell lines (REH, Nalm-6, and RS4;11), treatment with a combination of 50 nM RG7388 and 0.5 µM BBI608 resulted in a significant increase in p53 stabilization compared to either RG7388 alone (50 nM) or BBI608 alone (0.5 µM) (Figure 3C). This enhanced p53 stabilization was accompanied by a notable increase in p21^WAF1^ expression, indicative of the activation of the functional p53 pathway. p53 regulates the cellular stress response either by modulating the transcriptional activity of various proteins or by directly binding to the promoters of genes involved in apoptosis (such as *BAX*) or cell cycle arrest (such as *CDKN1A*, which encodes p21^WAF1^).

Densitometric analysis of the immunoblot bands shown in Figure 3C was performed for p53 and p21^WAF1^, with signal intensities normalized to GAPDH. A significant increase in both proteins was observed across the three ALL cell lines—REH, Nalm-6, and RS4;11—using paired *t*-tests (Figure 3D,E). A notable increase was detected in cells treated with a combination of 50 nM RG7388 and 0.5 μM BBI608 compared to 50 nM RG7388 alone. An increase in cPARP levels and a decrease in cMyc were also evident in the combination treatment group, as shown in the graph (Figure 3F,G), although this difference did not reach statistical significance.

### 2.4. BBI608 Enhances the mRNA Expression of P53 Transcriptionally Regulated Genes When Combined with the MDM2 Inhibitor RG7388

To investigate the hypothesis that combined treatment enhances p53 transcriptional activity in ALL cells, we assessed the mRNA expression levels of several candidate genes associated with cell cycle arrest and apoptosis using qRT-PCR. Specifically, we examined the expression of seven known p53 target genes in four ALL cell lines following treatment. The cells were treated with RG7388 at a concentration corresponding to 1×IC50 for each cell line, or with 0.5 μM BBI608. Both drugs were tested individually and in combination, with all treatments compared to a DMSO control group.

**Figure 4:**
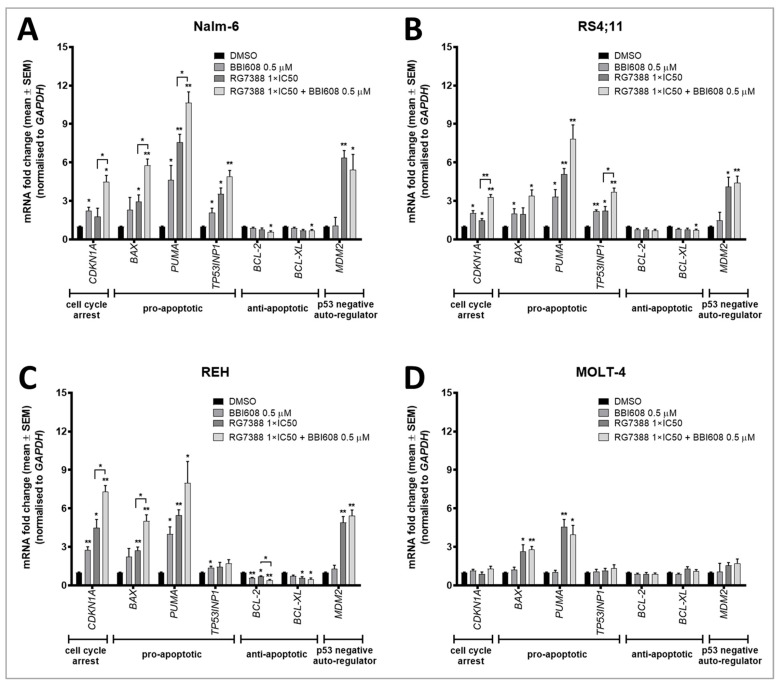
mRNA expression fold change in p53 transcriptional target genes by qRT–PCR. The mRNA expression levels of p53 transcriptionally regulated genes were measured in response to either 0.5 μM of the STAT3 inhibitor BBI608, the corresponding 1×IC50 concentration of RG7388 for each cell line, or combinations of both treatments for 24 h. Expression levels were compared relative to DMSO solvent and GAPDH control in Nalm-6 (**A**), RS4;11 (**B**), REH (**C**), and MOLT-4 (**D**) ALL cells. Statistical significance of differences were assessed using a paired *t*-test (* *p* < 0.05; ** *p* < 0.01) and indicated above each bar for each treatment compared with the DMSO control. The significance of differences with or without BBI608 is indicated above the horizontal bars. Only *p*-values < 0.05 are shown. Data are presented as the mean ± SEM from three independent experiments.

Our results revealed that RG7388 treatment significantly increased the expression of several p53 downstream target genes in p53-functional cells. Notably, *CDKN1A* (encodes p21), a key regulator of cell cycle arrest, and *MDM2*, involved in the p53-MDM2 auto-regulatory feedback loop, were both markedly upregulated in response to RG7388 (Figure 4A–C). In *TP53*^WT^ ALL cell lines, Nalm-6 and RS4;11, as well as in the *TP53*^MUT^ but functional REH cells, RG7388-mediated MDM2 inhibition resulted in an upregulation of p53 target genes (Figure 4A–C). However, no significant changes in gene expression were observed in the p53-non-functional MOLT-4 cells following RG7388 treatment.

In p53-functional cell lines, treatment with RG7388 at 1×IC50 for 24 h induced a significant upregulation of three pro-apoptotic genes (≥2-fold increase above baseline). Specifically, *PUMA* was upregulated by a mean of 6.6-fold, *BAX* by 2.6-fold, and *TP53INP1* by 2.1-fold. In contrast, only a slight downregulation of anti-apoptotic genes *BCL-2* and *BCL-XL* was observed, with pro-apoptotic gene induction being more prominent.

When combined with BBI608, a more pronounced induction of *CDKN1A* expression was observed, suggesting that the combination treatment enhanced cell cycle arrest and the p53-MDM2 feedback loop in ALL cells (Figure 4A–C). This enhancement was more robust than when each drug was applied individually. Additionally, a notable increase in the expression of pro-apoptotic genes *BAX* and *PUMA* was observed with the combined treatment, compared to either RG7388 or BBI608 treatment alone. The increased expression of these pro-apoptotic genes further supports the role of RG7388 and BBI608 in promoting apoptosis.

Interestingly, the expression of anti-apoptotic genes *BCL-2* and *BCL-XL*, which showed partial downregulation with single-agent treatments, was significantly further reduced with the combination treatment. In all three cell lines tested, this decrease was statistically significant, with a mean 1.8-fold decrease in *BCL-2* and 1.6-fold decrease in *BCL-XL* compared to untreated controls (Figure 4A–C).

In the MOLT-4 cell line, which is mutant for *TP53* and did not show a functional p53 response to RG7388 in Western blot analysis, we evaluated the transcriptional activity of p53 downstream target genes following both single and combination treatments. Unlike the other cell lines, RG7388 treatment alone did not induce any significant changes in gene expression in MOLT-4 cells (Figure 4D). However, BBI608 treatment alone resulted in a notable increase in the pro-apoptotic genes *BAX* and *PUMA* (Figure 4D). With combined treatment, there was no significant difference in the upregulation of these genes compared to single-agent BBI608 treatment (Figure 4D).

### 2.5. Combined Treatment with BBI608 and RG7388 Enhanced Apoptotic Responses, Demonstrated by an Increase in Annexin-Positive Cells

**Figure 5:**
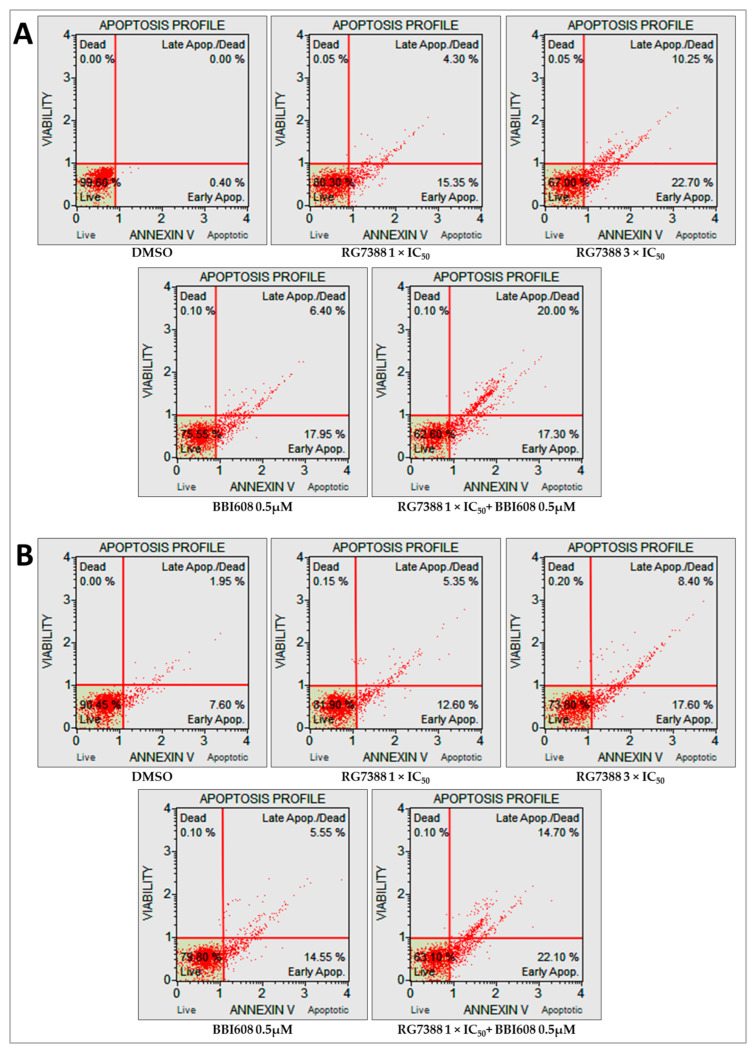
Detection of early apoptotic and late apoptotic/necrotic cells by annexin V/7-AAD staining in RS4;11 (**A**) and REH (**B**) cell lines. Cells were treated for 24 h with the corresponding 1×IC50 RG7388, 3×IC50 RG7388, 0.5 µM BBI608, or a combination of 1×IC50 RG7388 + 0.5 µM BBI608 for each cell line. Apoptotic cell percentages were analyzed using the Guava^®^ Muse^®^ Cell Analyzer (Cytek Biosciences, Fremont, CA, USA). The lower left quadrants indicate viable cells (annexin V-negative/7-AAD-negative), the lower right quadrants represent cells in the early stage of apoptosis (annexin V-positive/7-AAD-negative), and the upper right quadrants denote cells in the late stage of apoptosis or necrosis (annexin V-positive/7-AAD-positive). The percentages of the cell populations within each quadrant are shown.

Immunoblot analysis revealed visual evidence of a relative increase in cleaved PARP (cPARP) levels in p53-functional ALL cells following 24 h of treatment with RG7388 and BBI608, although this change did not reach statistical significance. Additionally, mRNA expression of p53-transcriptionally regulated pro-apoptotic genes demonstrated significant upregulation upon treatment with either MDM2 or STAT3 inhibitors, both individually and in combination. To further elucidate the apoptotic effects of RG7388, both as a monotherapy and in combination with BBI608, flow cytometry was performed utilizing annexin V and 7-AAD staining. The intensity of 7-AAD staining was plotted against annexin V staining to distinguish between viable (annexin V-negative/7-AAD-negative), early apoptotic (annexin V-positive/7-AAD-negative), and late apoptotic or necrotic (annexin V-positive/7-AAD-positive) cells. In matrix-based viability assays, RS4;11 and REH cell lines, both of which demonstrated synergistic responses to the combination treatment, exhibited increased proportions of annexin V and/or 7-AAD positive cells following single-agent RG7388 exposure, indicating apoptosis induction. This increase was statistically significant in both cell lines at 3×IC50 concentrations of RG7388. The combination of 1×IC50 RG7388 with 0.5 µM BBI608, in these same cell lines, enhanced the overall percentage of apoptotic cells compared to either agent alone (Figure 5A,B).

**Figure 6:**
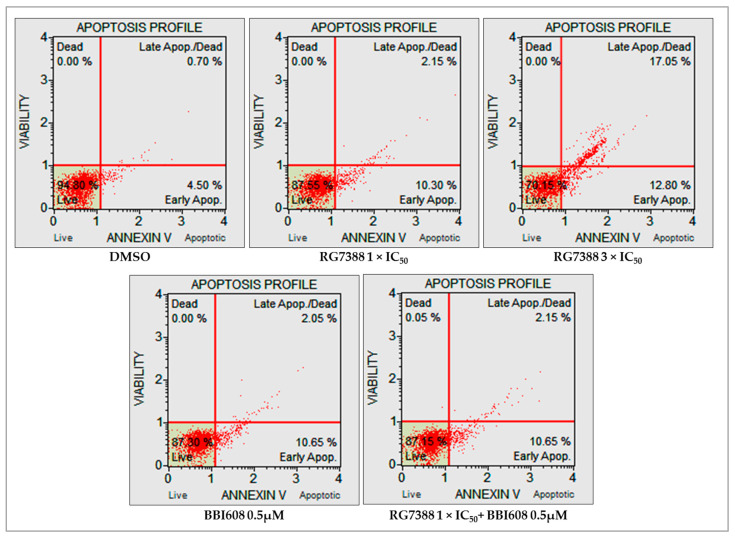
Detection of early apoptotic and late apoptotic/necrotic cells by annexin V/7-AAD staining in MOLT-4 cells. Cells were treated for 24 h with the corresponding 1×IC50 RG7388, 3×IC50 RG7388, 0.5 µM BBI608, or a combination of 1×IC50 RG7388 + 0.5 µM BBI608 for each cell line. Apoptotic cell percentages were analyzed using the Guava^®^ Muse^®^ Cell Analyzer. The lower left quadrants indicate viable cells (annexin V-negative/7-AAD-negative), the lower right quadrants represent cells in the early stage of apoptosis (annexin V-positive/7-AAD-negative), and the upper right quadrants denote cells in the late stage of apoptosis or necrosis (annexin V-positive/7-AAD-positive). The percentages of the cell populations within each quadrant are shown.

**Figure 7:**
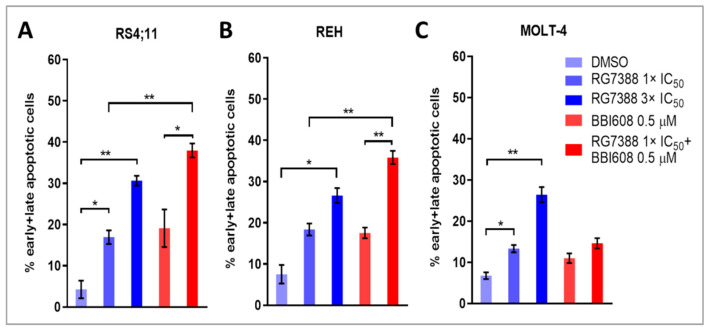
Changes in the percentages of early apoptotic and late apoptotic/necrotic cells in response to drug treatment in RS4;11 (**A**), REH (**B**), and MOLT-4 (**C**) cell lines. Cells were incubated separately with 1×IC50 RG7388, 3×IC50 RG7388, or 0.5 µM BBI608 for 24 h as single agents. Additionally, a combination of 1×IC50 RG7388 with 0.5 µM BBI608 was applied. The percentages of early and late apoptotic cells were measured by flow cytometry following annexin V/7-AAD staining. Data are expressed as mean ± SEM. Statistically significant differences between concentrations were determined using a paired sample *t*-test (* *p* < 0.05; ** *p* < 0.01).

In MOLT-4 cells, which did not exhibit significant synergy or antagonism in the combination index assays, RG7388 treatment at 1×IC50 and 3×IC50 concentrations alone induced a substantial increase in apoptosis. However, the addition of BBI608 did not lead to a further increase in apoptosis compared to RG7388 monotherapy (Figure 6). Figure 7 presents the statistical significance of the changes observed in the proportions of early apoptotic and late apoptotic/necrotic cells following drug treatment in RS4;11, REH, and MOLT-4 cell lines.

## 3. Discussion

ALL continues to pose a significant therapeutic challenge, particularly in high-risk populations such as those with *TP53* mutations or Philadelphia chromosome-positive (Ph+) ALL. Despite the significant strides that have been made in treatment modalities, including chemotherapy, radiotherapy, and targeted therapies, relapse remains frequent, and the limited efficacy of conventional treatments in adults emphasizes the urgent need for new therapeutic strategies. Targeting the p53 pathway through MDM2 inhibition and inhibiting STAT3, a key player in cancer cell proliferation, holds potential as an innovative approach to ALL therapy. This study explores the combined application of the MDM2 inhibitor (RG7388) and the STAT3 inhibitor (BBI608) to assess their potential to enhance apoptosis and inhibit tumor growth in ALL cells. Notably, both RG7388 and BBI608 are well-established, target-specific agents with demonstrated safety profiles. In particular, our prior study showed that RG7388 exhibits negligible cytotoxicity in healthy donor–derived PBMCs and hematopoietic stem cells [49], and BBI608, a natural naphthoquinone whose phytochemical origin may further support its low toxicity [81], has likewise been investigated across multiple monotherapy [82] and combination [83,84] clinical trials for its favorable tolerability and low dose-limiting or unexpected toxicities in malignant settings.

STAT3 exerts transcriptional control over the *TP53* gene through direct promoter engagement, wherein phosphorylated STAT3 dimers bind specific GAS (gamma-activated sequence) elements to suppress *TP53* transcription and attenuate the initiation of DNA damage-induced cell cycle arrest and apoptosis [74]. Persistent STAT3-driven upregulation of MDM2 further destabilizes p53 protein via enhanced ubiquitination and proteasomal degradation, establishing a feed-forward circuit that exacerbates p53 inactivation [85]. In hematological malignancies such as ALL, aberrant STAT3 activation often coincides with p53 pathway attenuation, and a recent study demonstrated that pharmacological or genetic STAT3 inhibition leads to induction of the p53 response in B-ALL cells [86], demonstrating the therapeutic potential of targeting this axis. These findings highlight the dual role of STAT3 as both a transcriptional repressor of p53 and an indirect facilitator of p53 turnover, positioning STAT3 inhibition as a powerful strategy to reinstate p53-dependent tumor-suppressive responses; our combined targeting of MDM2 and STAT3 with RG7388 and BBI608 synergistically enhanced p53 stabilization and downstream apoptosis in leukemia cell lines, validating this dual-target approach. Recent studies have also demonstrated the therapeutic potential of dual-targeted strategies in hematologic malignancies, further supporting the rationale for our combination approach in ALL [55,86,87].

Rather than merely confirming prior mechanistic assumptions, our study extends current understanding of the p53-STAT3 axis by demonstrating the functional interplay between these two pathways in the context of ALL. The combination of RG7388 and BBI608 produced synergistic effects in all p53^WT^ cell lines and in the p53^MUT^ cell line REH, which carries a heterozygous *TP53* mutation. The enhanced effectiveness of the combination therapy in REH highlights the potential of targeting both the MDM2 and STAT3 pathways in patients with partial p53 functionality. This finding is particularly significant as it suggests that combination treatments with MDM2 and STAT3 inhibitors could be applicable to a broader patient population, including those with partial p53 activity, thus offering new therapeutic opportunities for p53 mutant populations. The synergy observed in the REH cell line could be attributed to the partial functionality of p53, which may still allow for some degree of tumor suppressor activity in response to combined MDM2 and STAT3 inhibition. Previous studies have demonstrated that mutations at codon 181 do not entirely disrupt p53 function. Instead, these mutations retain the ability to transactivate *CDKN1A* and *MDM2* at levels similar to the wild-type p53 [88]. This aligns with the growing recognition that *TP53* mutations exist along a functional continuum, ranging from complete loss-of-function to dominant-negative and partial-function variants, each with distinct biological and clinical implications [89,90]. Partial-function mutations, such as the heterozygous codon 181 variant [90] in REH cells, may retain sufficient transcriptional activity to respond to p53-restorative therapies. Therefore, characterizing *TP53* mutation type and function may be critical for predicting treatment responsiveness and guiding personalized therapy in ALL. This finding builds upon existing research suggesting that p53 mutations do not exert the same effect across all genetic contexts, as their impact on function can vary in degree [91].

The selective ineffectiveness of the combination therapy in MOLT-4 cells underlines the requirement of at least partial p53 function for this dual-targeted approach to achieve synergy. This is consistent with the concept that MDM2 inhibition is futile in the absence of a functional p53 protein, a point reinforced by preclinical and clinical studies in hematologic malignancies and solid tumors. Although STAT3 inhibition alone retains some pro-apoptotic activity, as previously shown in studies involving STAT3-dependent leukemias [76,92,93], its contribution to the synergistic effect is clearly potentiated by a functional p53 axis. This specificity not only enhances therapeutic precision but also emphasizes the necessity for stratifying patients based on p53 functionality when considering combination regimens.

Furthermore, our results align with a growing body of literature indicating that oncogenic STAT3 activation contributes to immune evasion, chemoresistance, and disease persistence in leukemia [94,95,96,97]. By targeting STAT3, BBI608 may not only sensitize cells to MDM2 inhibition but also potentially modulate the tumor microenvironment, although this was not directly assessed in our in vitro model. Future studies incorporating co-culture systems or in vivo models may help elucidate this broader immunomodulatory role.

The pronounced upregulation of p53 target genes and the pro-apoptotic shift in gene expression observed following combination treatment provide compelling evidence of reactivated tumor suppressor activity. These gene expression changes are consistent with the literature on p53-dependent apoptosis pathways, including transcriptional activation of *CDKN1A*, *BAX*, and *PUMA*, and suppression of anti-apoptotic mediators like *BCL-2* and *BCL-XL*. Importantly, our flow cytometry-based apoptosis assays reinforce this transcriptional data, indicating an apoptotic response that is significantly enhanced by dual pathway inhibition in p53-proficient settings. This integrated response suggests that dual inhibition acts not merely through additive toxicity, but by dismantling a pathogenic regulatory circuit that simultaneously stabilizes p53 and blocks its upstream repression.

In this study, we demonstrate that pharmacological inhibition of STAT3 using BBI608 leads to reduced c-Myc expression and enhanced p53 stabilization, supporting its role as a key oncogenic driver in ALL. However, STAT3 functions as a pleiotropic transcription factor involved in multiple oncogenic and immunomodulatory processes beyond c-Myc regulation. Its activity is tightly regulated by phosphorylation at Tyr705, which enables dimerization and nuclear translocation, and at Ser727, which modulates transcriptional activity [98]. These phosphorylation events are commonly mediated by upstream kinases such as JAKs, SRC family kinases, and MAPKs, integrating diverse extracellular cues, including cytokine signaling [99,100]. Although the present study did not directly assess STAT3 phosphorylation status or upstream kinase activation, the observed anti-leukemic effects of BBI608 may reflect broader disruption of these regulatory nodes. Moreover, STAT3 is known to repress p53 both transcriptionally and post-translationally [74], and our data suggest that simultaneous STAT3 and MDM2 inhibition may act through complementary mechanisms to restore p53 activity. Future studies employing phospho-specific assays and pathway-level analyses will be essential to dissect the full spectrum of STAT3-mediated signaling in ALL and to further validate the mechanistic synergy observed in our combination strategy. Similar combinatorial approaches targeting oncogenic signaling nodes, including MAPK and AKT pathways, have also shown promise in solid tumors such as colorectal cancer, further supporting the translational relevance of dual-targeted strategies [101].

Despite these promising findings, some limitations warrant consideration. First, our study was conducted exclusively in vitro using established ALL cell lines, which may not fully recapitulate the heterogeneity and complexity of patient-derived leukemic cells or the influence of the bone marrow microenvironment. The absence of in vivo data limits the translational scope of our results, particularly in assessing drug bioavailability, pharmacokinetics, and immune-mediated effects. Another limitation is the use of static end-point assays to measure apoptosis and gene expression; future studies employing time-course analyses or single-cell approaches could yield more nuanced insights into dynamic treatment responses. Finally, although our findings suggest a clear dependency on functional p53 for synergistic effects, the potential off-target activities of BBI608 and RG7388 were not explored in depth and should be considered in further mechanistic studies or combination screens. Future studies will include the use of patient-derived leukemic cells to validate the observed effects in a clinically relevant ex vivo setting, as well as the development of xenograft mouse models to assess the in vivo efficacy, pharmacokinetics, and safety profile of the RG7388-BBI608 combination. These approaches will also allow for evaluation of interactions with the tumor microenvironment, including immune-mediated effects, thereby bridging the gap between in vitro findings and clinical applicability.

As with all combinatorial therapies, the translation of RG7388 and BBI608 into clinical use for ALL will require careful consideration of pharmacological factors that may impact safety and efficacy. Although both agents have shown favorable safety profiles in monotherapy trials [82,102], co-administration may introduce new complexities. For example, RG7388 is known to cause dose-dependent gastrointestinal and hematologic toxicities such as thrombocytopenia [103], while BBI608 has been associated with diarrhea and fatigue in clinical studies [84,104]. Although these toxicities are generally manageable, their potential overlap warrants close monitoring in combination settings. Moreover, the pharmacokinetics of each drug may differ: RG7388 is a highly protein-bound, orally available MDM2 inhibitor metabolized via CYP3A4 [105], while BBI608 is extensively metabolized primarily via reductive pathways before excretion predominantly through fecal and urinary routes [106]. These differences may affect drug exposure and necessitate optimization of dosing regimens. Additionally, resistance mechanisms—such as p53 mutation acquisition for RG7388 or STAT3 bypass signaling for BBI608—could potentially compromise long-term response when used together. Finally, possible pharmacodynamic interactions with other agents commonly used in ALL regimens, such as glucocorticoids [107] or tyrosine kinase inhibitors [9], should be systematically investigated.

## 4. Materials and Methods

### 4.1. Cell Lines and Culture Conditions

The human acute lymphoblastic leukemia cell lines Nalm-6, HAL-01, REH, MOLT-4, RS4;11, and CCRF-CEM were obtained from authenticated cell line repositories (ATCC or DSMZ). These cell lines were cultured in RPMI-1640 medium (Gibco, Waltham, MA, USA), supplemented with 10% fetal bovine serum (FBS) (Gibco, Waltham, MA, USA) and 100 U/mL penicillin/streptomycin (Sigma-Aldrich, St. Louis, MO, USA) in appropriate culture vessels (Labselect, Hefei, China). The cells were maintained at 37 °C in a CO_2_ incubator (Esco, St. Louis, MO, USA) with 5% CO_2_. Cell viability, morphology, and contamination were monitored on a daily basis. The panel of cell lines included three *TP53* mutant and three wild-type *TP53* cell lines (as detailed in Table 1). Specifically, the REH cell line carries a missense mutation at codon 181, while the CCRF-CEM cell line harbors missense mutations at codons 175 and 248 [108]. In contrast, the MOLT-4 cell line presents a nonsense mutation at codon 306 [109], leading to a premature stop codon. These mutations affect the DNA-binding domain of p53 in both REH and CCRF-CEM cells, while in MOLT-4 cells, the mutation generates a stop codon in the nuclear localization domain of the protein [110].

### 4.2. Compounds

RG7388 (MDM2 inhibitor, also known as idasanutlin) and BBI608 (STAT3 inhibitor, also known as napabucasin) were used in this study. Both inhibitors, obtained as powders (Cayman #21532 for RG7388 and Cayman #22255 for BBI608), were dissolved in DMSO (dimethyl sulfoxide) (Serva #20385.02) to prepare 10 mM stock solutions, stored at −20 °C. Prior to each experiment, serial dilutions were made in DMSO to achieve the desired concentrations, ensuring that DMSO did not exceed 0.5% (*v/v*) in the culture medium to minimize cytotoxic effects.

### 4.3. Cell Viability Assay

Cells were seeded in 96-well plates (Labselect) at a density of 100 µL complete medium per well, 24 h prior to inhibitor treatment. The optimal starting cell density of 2 × 10^5^ cells/mL, determined from growth curves of each cell line, allowed for at least two doubling times and was used in subsequent XTT assays. On the following day, cells were treated with RG7388 and BBI608, either individually or in combination, at a final DMSO concentration of 0.5%, and incubated for 72 h. Cell viability was assessed using the XTT assay (BioInd #BI20-300-1000), which measures mitochondrial activity [111]. The percentage of viability was calculated by comparing treated cells to DMSO control wells. The half-maximal inhibitory concentration (IC50) values for both inhibitors were determined through at least three independent experiments, with IC50 representing the concentration required to reduce mitochondrial activity by 50%.

### 4.4. Assessment of Combination Treatment

The combination of RG7388 and BBI608 was evaluated in *TP53*- and *STAT3*-status defined ALL cell lines. Each inhibitor was tested individually (RG7388 or BBI608) and in combination (RG7388 + BBI608) for 72 h. Combination effects were assessed using concentration-response matrix analysis, with doses determined based on IC50 values derived from individual drug experiments. Synergy was analyzed using the SynergyFinder web application, employing the Zero Interaction Potency (ZIP) model to evaluate drug interactions [112,113]. The effects were classified as synergistic, antagonistic, or additive based on the observed versus expected responses. ZIP analysis compares changes in concentration-response curves to measure the strength of drug interactions.

### 4.5. Immunoblotting

Cells were cultured at a density of 1 × 10^6^ cells/mL in 2 mL of culture medium per well in 6-well plates (Labselect) and treated with the specified doses of inhibitors, either individually or in combination. After 24 h, protein lysates were harvested using lysis buffer (Takara Bio Europe, Saint-Germain-en-Laye, France) and heated at 100 °C for 10 min. Protein concentrations were quantified using the BCA Protein Assay Kit (Takara Bio Europe, Saint-Germain-en-Laye, France). Equal amounts of protein were loaded onto 4–20% pre-cast SDS-PAGE gels (Biorad, Gen Era Diagnostik, İstanbul, Türkiye) and separated by electrophoresis using the Biorad Mini-PROTEAN Tetra Cell system (Gen Era Diagnostik, İstanbul, Türkiye). The proteins were subsequently transferred to a PVDF membrane (Biorad, Gen Era Diagnostik, İstanbul, Türkiye) via a wet transfer system (Cleaver Scientific, Warwickshire, UK).

Primary antibodies used for detection included p53 (DO-7, CST), p21^WAF1/CIP1^ (CST #12D1), PARP/cPARP (CST #46D11), STAT3 (CST #9139), cMyc (CST #5605), and GAPDH (Sigma, Kanagawa, Japan). Cleaved PARP (cPARP) is a well-established marker of apoptosis. During programmed cell death, effector caspases such as caspase 3 and caspase 7 cleave PARP1 at a specific DEVD site, generating an approximately 89 kDa fragment. This cleavage inactivates PARP1 and prevents unnecessary DNA repair, facilitating the apoptotic process [114]. The p53 and p21^WAF1/CIP1^ antibodies were used to evaluate the molecular response to MDM2 inhibition. Stabilization of p53 represents a key marker of target engagement, whereas upregulation of p21 reflects functional p53 transcriptional activity, serving as evidence of pathway activation [115]. The c-Myc antibody was used to evaluate the downstream effects of STAT3 inhibition. As a direct transcriptional target of STAT3, reduced c-Myc expression serves as a functional indicator of effective pathway suppression [116].

HRP-conjugated secondary antibodies (CST #7076S and #7074S) were applied for signal detection. Chemiluminescent signals were captured using the Biorad Clarity Western ECL reagent (Gen Era Diagnostik, İstanbul, Türkiye) and imaged with the Syngene G:Box platform. Densitometric analysis of immunoblot bands was performed using the freely available ImageJ software (version 1.53t; National Institutes of Health, Bethesda, MD, USA). The intensity of each target protein band was quantified and normalized to the corresponding GAPDH signal, which served as a housekeeping control to account for loading variability.

### 4.6. RNA Extraction and Quantitative Real-Time PCR (qRT-PCR) Analysis

Cells were cultured at a density of 1 × 10^6^ cells/mL in 12-well plates (Labselect) and treated with RG7388 or BBI608, either alone or in combination, for 24 h. Total RNA was extracted using the Aurum™ Total RNA Mini Kit (Biorad, Gen Era Diagnostik, İstanbul, Türkiye). RNA quality and concentration were assessed using a spectrophotometer. Complementary DNA (cDNA) was synthesized using the Promega reverse transcriptase kit (Promega Corporation, Madison, WI, USA). Quantitative real-time PCR (qRT-PCR) was performed using SYBR Green Master Mix (Life Technologies, Thermo Fisher Scientific, Waltham, MA, USA) according to the manufacturer’s instructions. Each qRT-PCR reaction was conducted with 20 ng of cDNA in a final reaction volume of 10 μL, utilizing standard cycling conditions. Products were detected in real time on a Roche Light Cycler 480^®^. PCR primers (Table 3) were designed based on cDNA sequences (GenBank™) to evaluate changes in gene expression. Data were analyzed using the 2^−ΔΔCT^ method, with gene expression normalized to the *GAPDH* housekeeping gene and compared to the corresponding untreated (DMSO control) samples. A no-template control was used to control for contamination of external DNA in reactions.

### 4.7. Apoptosis Analysis by Annexin V/7-AAD Using Fluorescence-Activated Cell Sorting

Fluorescence-activated cell sorting (FACS) analysis was employed to assess apoptosis. Cells were cultured at a density of 2.5 × 10^5^ cells/mL in 6-well plates (Labselect) and treated with RG7388 and BBI608, either individually or in combination, for 24 h. Following treatment, cells were collected, washed with cold Dulbecco’s Phosphate-Buffered Saline (D-PBS), and resuspended in cold D-PBS for analysis. Apoptosis was evaluated using the Muse^®^ Annexin-V and Dead Cell Kit (Cytek Biosciences, Fremont, CA, USA), which detects phosphatidylserine (PS) exposure on the external membrane of apoptotic cells and assesses membrane integrity. Four distinct cell populations were identified: live cells (annexin V-negative/7-AAD-negative), early apoptotic cells (annexin V-positive/7-AAD-negative), late apoptotic and dead cells (annexin V-positive/7-AAD-positive), and necrotic cells (annexin V-negative/7-AAD-positive). Flow cytometry analysis was performed using the Guava^®^ Muse^®^ Cell Analyzer (Cytek Biosciences, Fremont, CA, USA).

### 4.8. Statistical Analysis

Statistical analysis and graph generation were conducted using GraphPad Prism software (version 8.0.1). Unpaired *t*-tests were employed for statistical comparisons based on predefined hypotheses between specific pairs of groups, unless stated otherwise. Due to the limited number of comparisons and the hypothesis-driven design, no formal correction for multiple comparisons was applied. Data are presented as means ± standard error of the mean (SEM). A *p*-value of <0.05 was considered statistically significant. Statistical significance was defined as: * *p* < 0.05, ** *p* < 0.01, *** *p* < 0.001, **** *p* < 0.0001.

## 5. Conclusions

In conclusion, this study highlights the promising therapeutic potential of combining MDM2 inhibition with STAT3 inhibition in ALL, particularly for p53^WT^ and p53^MUT^ cells with at least partial p53 functionality. The synergistic effects observed in p53^WT^ and p53^MUT^ cells with partial p53 functionality suggest that dual inhibition of MDM2 and STAT3 effectively enhances cell death mechanisms, providing a compelling rationale for this combination therapy. This approach could benefit a broader patient population, including those with partial p53 mutations, thereby expanding its therapeutic applicability.

Moreover, the lack of efficacy in certain p53 mutant cell lines emphasizes the importance of considering both p53 and STAT3 status when developing treatment strategies and highlights the need for new therapeutic targets in patients with diverse mutation spectra. Our study contributes to the growing body of evidence supporting the potential of MDM2 inhibitors, such as RG7388, to restore p53 functionality and promote apoptosis in cancer cells. Moving forward, further preclinical and clinical studies are necessary to validate the safety and efficacy of these combination therapies and to identify predictive biomarkers for patient responses. The combined inhibition of MDM2 and STAT3 presents a promising therapeutic strategy that warrants continued exploration in both ALL and other cancers characterized by p53 deficiency or STAT3 dysregulation.

## Figures and Tables

**Table 1:** Genetic profiles and IC50 values of RG7388 and BBI608 for the cell lines used in the study. All cell lines are of ALL (B or T ALL) origin. The table summarizes the genetic characteristics of each cell line along with the IC50 values for RG7388 and BBI608.

Cell Lines	Cell Type	* *TP53* Status	* *STAT3* Status	** RG7388 (nM)	** BBI608 (nM)
Nalm-6	B cell ALL	WT	WT	82 ± 6	562 ± 43
RS4;11	B cell ALL	WT	WT	47 ± 8	659 ± 79
HAL-01	B cell ALL	WT	WT	50 ± 11	702 ± 18
MOLT-4	T cell ALL	Mutant (Heterozygous) c.916C>T; p.R306 *	Mutant (Heterozygous) c.2186G>A; p.R729H c.1217C>T; p.A406V	52 ± 11	1114 ± 226
REH	B cell ALL	Mutant (Heterozygous) c.541C>T; p.R181C	WT	289 ± 9	811 ± 154
CCRF-CEM	T cell ALL	Mutant (Heterozygous) c.524G>A; p.R175H c.743G>A; p.R248Q	WT	>3000	681 ± 45

* *TP53* and *STAT3* gene mutational status were taken from COSMIC (Catalogue of Somatic Mutations in Cancer) database. ** The IC_50_ values shown represent the mean of at least *n* = 3 independent repeats ±SEM. WT: wild-type; nM: nanomolar; ALL: acute lymphoblastic leukemia.

**Table 2:** Summary of combined treatment synergy scores for ALL cell lines. The table presents the global synergy scores (mean) across the entire dose matrix, as well as the synergy score for the most synergistic area.

Cell Lines	* Global Synergy Score	Most Synergistic Area Score	** Multi-Dimensional Synergy of Combinations (MuSyC) Reference Model
Nalm-6	13.9	24.4	There is a synergistic potency shift induced by RG7388.
RS4;11	13.6	25.7	There is a synergistic potency shift induced by RG7388.
REH	11.6	13.0	There is a synergistic potency shift induced by BBI608. There is a positive cooperativity induced by RG7388.
HAL-01	8.5	13.5	There is a synergistic potency shift induced by BBI608.
MOLT-4	1.2	9.9	No synergy or antagonism detected with 95 percent confidence interval.
CCRF-CEM	2.3	14.6	No synergy or antagonism detected with 95 percent confidence interval.

* The delta (δ) synergy score by using Zero Interaction Potency (ZIP) method less than −10: the interaction between two drugs is likely to be *antagonistic*; from −10 to 10: the interaction between two drugs is likely to be *additive*; larger than 10: the interaction between two drugs is likely to be *synergistic*. ** MuSyC reference model explores whether the observed synergy is due to enhanced potency and/or efficacy or both (cooperativity) of the single agents.

**Table 3:** Forward and reverse primer sequences used for quantitative real-time PCR.

Genes	Forward Primers (5′-3′)	Reverse Primers (5′-3′)
*GAPDH*	CGACCACTTTGTCAAGCTCA	GGGTCTTACTCCTTGGAGGC
*PUMA (BBC3)*	ACCTCAACGCACAGTACGA	CTGGGTAAGGGCAGGAGTC
*MDM2*	AGTAGCAGTGAATCTACAGGGA	CTGATCCAACCAATCACCTGAAT
*CDKN1A*	TGTCCGCAGAACCCATGC	AAAGTCGAAGTTCCTCGCTC
*TP53INP1*	TCTTGAGTGCTTGGCTGATACA	GGTGGGGTGATAAACCAGCTC
*BAX*	CCCGAGAGGTCTTTTTCCGAG	CCAGCCCATGATGGTTCTGAT
*BCL-2*	GGTGGGGTCATGTGTGTGG	CGGTTCAGGTACTCAGTCATCC
*BCL2L1* *(BCL-XL)*	GCAGGCGACGAGTTTGAACT	CTCGGCTGCTGCATTGTT

## Data Availability

The original contributions presented in this study are included in the article. Further inquiries can be directed to the corresponding author.
